# Rayleigh–Bloch waves along elastic diffraction gratings

**DOI:** 10.1098/rspa.2014.0465

**Published:** 2015-01-08

**Authors:** D. J. Colquitt, R. V. Craster, T. Antonakakis, S. Guenneau

**Affiliations:** 1 Department of Mathematics, Imperial College London, London SW7 2AZ, UK; 2 80 Capital LLP, London W1S 4JJ, UK; 3 Institut Fresnel, UMR CNRS 7249, University of Aix–Marseille, Marseille, France

**Keywords:** plasmonics, homogenization, elastic waves

## Abstract

Rayleigh–Bloch (RB) waves in elasticity, in contrast to those in scalar wave systems, appear to have had little attention. Despite the importance of RB waves in applications, their connections to trapped modes and the ubiquitous nature of diffraction gratings, there has been no investigation of whether such waves occur within elastic diffraction gratings for the in-plane vector elastic system. We identify boundary conditions that support such waves and numerical simulations confirm their presence. An asymptotic technique is also developed to generate effective medium homogenized equations for the grating that allows us to replace the detailed microstructure by a continuum representation. Further numerical simulations confirm that the asymptotic scheme captures the essential features of these waves.

## 1. Introduction

For scalar wave systems such as those in linear water waves, acoustics, polarized electromagnetic, flexural waves or shear-horizontal polarized elastic waves, it has long been known that one can find a class of surface waves created by periodicity in the geometry of the surface or equivalently by a periodic diffraction grating embedded within the medium. These waves propagate along a surface, exponentially decay in amplitude perpendicular to the surface and are created by geometric periodic corrugations, or perturbations, to the surface [1–3] in situations where a surface wave would otherwise not exist. Although often studied for surface waves, they also propagate along diffraction gratings and provide a mechanism for energy transport and guiding along gratings [4] and arrays [5]. Owing to their ubiquitous nature in these different settings, they have been discovered and rediscovered several times and appear as edge waves [6] for water waves localized to periodic coastlines, spoof surface plasmon polaritons (SPPs) [7,8] in modern applications of plasmonics, array-guided surface waves [9] in Yagi–Uda antenna theory, Rayleigh–Bloch surface waves [10,11] for scalar wave systems with diffraction gratings, and in elastic plates [12,13] among other areas. The name Rayleigh–Bloch (RB hereinafter) waves seems most descriptive as surface waves are typically called Rayleigh waves and Bloch waves arise due to periodicity.

**Figure 1.**
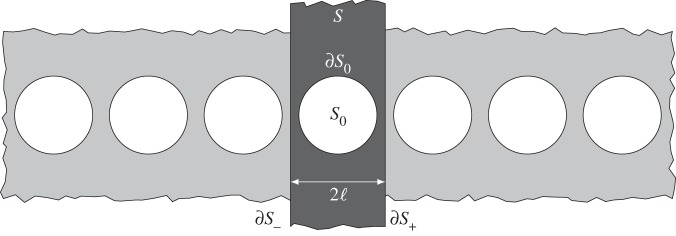
An array of cylindrical voids *S*_0_ with the elementary cell *S* highlighted in dark grey. The distance between the centres of two adjacent voids is 2ℓ and the cell is infinite in the vertical direction.

**Figure 2.**
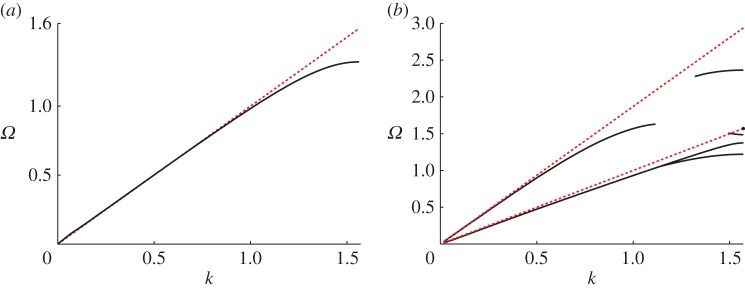
The dispersion curves for RB waves propagating along a linear array of cylindrical voids (cf. figure 1). Here, *Ω* is radian frequency and *k* is the Bloch wavenumber. (*a*) For the acoustic case (e.g. anti-plane shear waves) of an array of Neumann cylindrical voids; (*b*) for the elastic case (coupled in-plane shear and pressure waves) where the boundary conditions are traction free. The voids have radius 0.8 and ℓ=1 in both cases. The (red) dotted lines show the light-lines in each case. The reader’s attention is drawn to the different scales on the ordinate axes. We also emphasize that the lowest dotted line in (*b*) corresponds to in-plane shear waves, whereas the upper one which is for in-plane pressure waves is markedly different from that of acoustics waves in (*a*). (*a*) Acoustic case and (*b*) elastic case.

RB waves have been of particular interest in terms of their existence [14,15] as they require either Neumann boundary conditions upon the grating, or penetrable scatterers (see [16] among others), to exist and they have implications, as non-zero eigenfunctions of the system, in terms of uniqueness of solution and connection to trapped modes; the Dirichlet cases cannot support RB waves [10]. Curiously, given this substantial literature, the importance of RB waves in applications, their connections to trapped modes [17] and the universal nature of diffraction gratings [18] there has been no investigation of whether RB waves occur, or even exist, for elastic diffraction gratings for the in-plane vector elastic system; we address the diffraction grating here. For the scalar case of, say, an array of Neumann cylindrical voids [19,20] (figure 1) one finds that there is a dispersion relation connecting the phase shift along the array to the frequency and RB waves exist beneath the so-called light-line (the linear dispersion relation from the surrounding bulk medium; figure 2*a*). In the low-frequency limit, the RB dispersion curve approaches the bulk dispersion relation and in many situations, for instance, comb-like surfaces [2,6], one can have multiple RB waves beneath the light-line.

In isotropic linear elasticity, the bulk medium now supports two waves, compressional and shear, with differing wave speeds and therefore two light-lines exist (e.g. figure 2*b*), moreover the natural boundary conditions in elasticity for a realistic grating are either rigid or traction free; neither are simply Neumann or Dirichlet and the fields are coupled at these boundaries, that is, conversion of pressure (resp. shear) to shear (resp. pressure) waves occur at the boundaries. It is therefore not obvious whether the theory underlying scalar RB waves will translate to the elastic system. We identify the boundary conditions that can support such waves, numerical simulations confirm their presence and an asymptotic technique is developed to generate effective medium equations for them. It is worth noting that, recently, RB waves have been studied in the setting of the full Maxwell’s equations for electromagnetic waves propagating along a linear array of dielectric spheres [21]. Yet, despite several papers analysing scattering problems for arrays of spheres and cylinders in full vector elasticity (e.g. [22–26]), to the best of our knowledge there has been no study of the existence or behaviour of RB waves for the Lamé system.

We will couch our study in the language of homogenization theory as we can then simultaneously generate RB waves and an asymptotic theory for them. We shall view the diffraction grating, as shown in figure 1, as consisting of two length scales; a short-scale set by the periodicity and a long scale. The long-scale can be thought of as the overall size of the grating,^1^ or the typical macroscopic wavelength dominating the dynamic response (cf. §4*a*), or some other physical length that is large compared with the microscopic cell size. The precise physical nature of the long scale is unimportant for the analysis which follows; it is sufficient that there exists some length *L* such that ℓ/*L*≪1. This separation of scales is then a natural setting for multiple scales with a fast scale and slow scale in space and thus for homogenization. Classically, homogenization theory is developed for long waves that are of low frequency, and for bulk media (not surfaces) and is detailed in many monographs, [27–30], and essentially relies upon the wavelength being much larger than the microstructure which is usually assumed to be perfectly periodic: this limits the procedure to low frequencies/long waves and a quasi-static situation. For many applications, particularly in optics such as photonics, the limitation to low frequencies is a serious deficiency, nonetheless, the attraction of having an effective equation for a microstructured medium where one need no longer model the detail of each individual scatterer is highly attractive [31]. The classical theory has been recently extended in three directions: to higher frequencies where the high-frequency homogenization of [32] provides the necessary methodology (although limited to scalar waves) with short-scale structure within the solution captured by standing Bloch waves and this then modulated by a long-scale envelope function that satisfies a partial differential equation. The upshot of the theory is that an effective equation is deduced entirely on the long scale with the microstructure encapsulated by spatially dispersive coefficients (e.g. [33]). Recently, the theory has also been extended to in-plane elasticity for bulk media [34,35] and to scalar RB waves [20]. Therefore, the theory is ideally suited to the exploration of elastic RB waves.

We begin with a brief discussion of RB waves in §2, which is followed by a formulation of the problem in the asymptotic setting of high-frequency homogenization (§3). The asymptotic theory then leads naturally to an effective condition for the diffraction grating, as an ordinary differential equation on the long scale, that supports waves that travel along the array. These RB waves are then found numerically in §4 for an array of cylindrical voids that have a traction-free boundary condition upon them. Finally, some concluding remarks are drawn together in §5.

## 2. Rayleigh–Bloch waves

**Figure 3.**
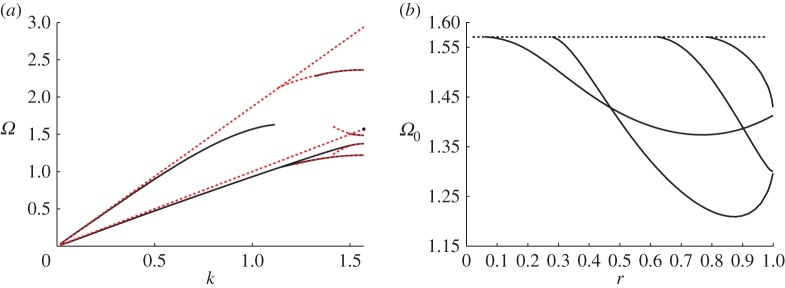
(*a*) The dispersion diagram for localized RB-like modes (i.e. modes that decay perpendicular to the grating such that |**u**|→0 as $|x_{2}|\to \infty$). The exact dispersion curves, obtained by solving the full problem numerically, are indicated by the solid (black) lines; while the asymptotics resulting from high-frequency homogenization are shown as dotted (red) lines. (*b*) The variation in standing wave frequencies, *Ω*_0_ with increasing radius, *r*. The dashed line in (*b*) indicates the intersection of the shear light-line with the line *k*=*π*/2.

As discussed earlier, RB waves have a long history for scalar wave systems that are equivalent to the Helmholtz equation. In the bulk medium, which is dispersionless, there is a linear relation between wavenumber and frequency; this linear bulk dispersion relation is the light-line shown in figure 2*a* as the dotted red line. For a periodic linear array, it is conventional to relate the phase shift along the array, characterized by the Bloch wavenumber (defined here by the Bloch–Floquet quasi-periodicity condition such that $\text{u}{|}_{\partial S_{-}}=\text{u}{|}_{\partial S_{+}}\;e^{i2k\ell }$ where ∂*S*_+_ denote the boundaries of the elementary cell as illustrated in figure 1) to frequency (*Ω*). In this paper, it is convenient to work with a non-dimensional Bloch wavenumber and to this end, we take the semi-width of the cell as a natural unit and set ℓ=1. To have eigensolutions that decay, as one tends to infinity in directions perpendicular to the array, one typically needs to be below the light-line to prevent coupling into radiation. There are some counterexamples to this, in particular, there are embedded RB waves [17] but we will not discuss them or their analogues in elasticity here. For a linear array of Neumann cylindrical inclusions, with radius 0.8, for the Helmholtz equation the RB dispersion curves are shown in figure 2*a*. Notably, as the radius increases further beyond 0.81, another RB mode emerges from the light-line and this feature is also seen in the elastic case; the standing wave frequencies (*Ω* when *k*=*π*/2 and, hence, **u**|_∂*S*_−__=−**u**|_∂*S*_+__) for the lowest four modes are shown as a function of radius in figure 3*b* with one emerging near 0.8.

A good intuitive guide to the possible existence of RB waves for an array or grating is to consider a doubly periodic rectangular array of inclusions, say, of fixed width, 2, in *x*_1_ and of height *d* in *x*_2_. The limit of *d*=2 is that of a doubly periodic array of cylinders in a square array and the limit of d→∞ is that of the linear array we consider here. In the scalar case, the square array is an oft-studied system [36] and the Dirichlet case has a zero frequency stop-band, that is, the dispersion curves lie above the light-line for *d*=2 and never cross beneath it as *d* increases. Unsurprisingly, therefore one observes no RB waves for a linear array of Dirichlet cylinders. Conversely, for a square array of Neumann cylinders there is a low-frequency linear dispersion relation through the origin, beneath the light-line, and as the radius increases an additional mode passes beneath the light-line—both remain as *d* increases. We now parallel this intuitive process for elastic inclusions: the two cases of primary interest are rigid inclusions or traction-free inclusions.

**Figure 4.**
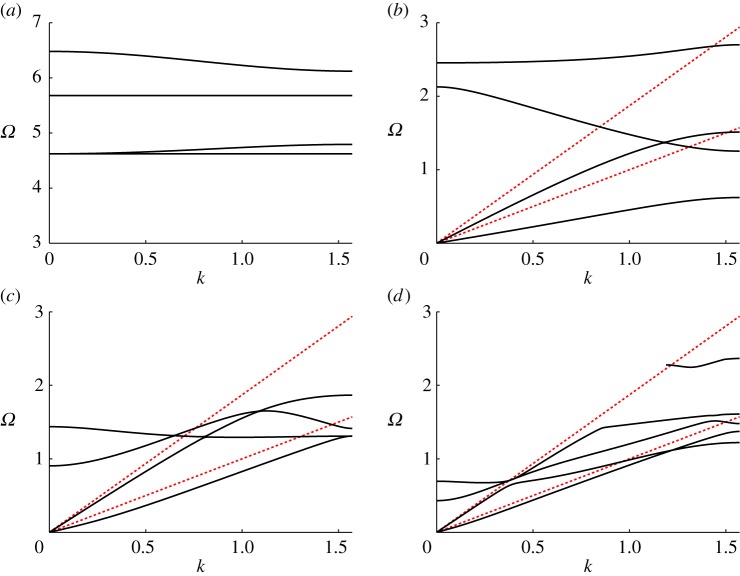
(*a*) The lowest four dispersion curves for a doubly periodic array of cylindrical rigid (clamped) voids for in-plane elasticity. (*b*–*d*) The same geometry, but for traction-free voids, for cells of dimension 2×2, 2×5 and 2×10, respectively. The voids have radius 0.8 in all cases and the two (shear and compressional) light-lines are indicated by the two (red) dotted lines. The reader’s attention is drawn to the different ordinate axis for (*a*) compared with (*b*) and (*c*).

On the microstructure, shown in figure 1, *S*_0_ represents the inclusion and we could set either traction-free or rigid conditions on its boundary (∂*S*_0_) depending on the physical setting under consideration; for the sake of clarity, we do not consider the case of penetrable scatters in the present paper. Figure 4*a* shows the first four dispersion curves for a doubly periodic array of rigid voids for two-dimensional in-plane elasticity (e.g. [35]). In this case, there is a zero frequency stop-band and, hence, we would not expect to see RB-type modes; this is analogous to the scalar Dirichlet case mentioned earlier.

On the other hand, parts (*b*)–(*d*) of the same figure show the corresponding curves for the same geometry, but for voids with traction-free boundaries, and for cells of increasing height *d*=2, 5 and 10, respectively. In these cases, we observe two acoustic modes emerging from the origin, both of which, eventually lie beneath the shear light-line in the vicinity of *k*=*π*/2 and that resemble the lowest two dispersion curves in figure 2*b*. These also have a similarity with the RB dispersion curve shown in the acoustic case (cf. figure 2*a*).

As the height of the elementary cell increases, that is, as the doubly periodic geometry approaches that of the diffraction grating, the higher frequency modes collapse down onto the shear light-line (which is the lower one), with a key difference between the elastic and acoustic cases, being the persistence of a RB mode near the compressional light-line as illustrated in figure 2*b*. The fifth mode can also be observed in figure 4*d* where the doubly periodic array of voids tends to the grating geometry.

It is notable that the numerical results were produced using the commercial finite-element software Comsol Multiphysics and that the infinite strip is created computationally using (elastic) perfectly matched layers; these are standard numerical approaches. There is, however, an issue with a large number of spurious modes detected that lie in-between the light-lines. We have carefully tracked the decaying RB modes and have left gaps in the third and fifth RB modes in figure 2*b* where the accuracy was diminished by the proximity of spurious modes. However, as we shall see, the asymptotic method developed herein will allow us to extract all the salient information regarding the behaviour of RB waves despite the numerical difficulties associated with the full computation.

## 3. Diffraction gratings: an asymptotic analysis

The time-harmonic deformation of a linearly elastic solid is governed by the elastodynamic wave equation [37]

3.1 $$\sigma_{ij,j}+\rho \omega^{2}u_{i}=0,\;\mathrm{for}\;i,j=1,2,$$

where repeated indices are summed over hereafter unless otherwise stated and subscript commas followed by lowercase letters indicate differentiation with respect to the spatial variables. The stresses are denoted by *σ*_*ij*_, displacements by *u*_*i*_, and the density and angular frequency are *ρ* and *ω*, respectively. We choose to use a Cartesian coordinate frame, *x*_*i*_, with the diffraction grating orientated in the *x*_1_ direction. As we assume homogeneous isotropic linearly elastic media, the constitutive equation relating stress and strain is

3.2 $$\sigma_{ij}=\lambda u_{k,k}\delta_{ij}+\mu (u_{i,j}+u_{j,i}),$$

with *λ*,*μ* as the Lamé coefficients and *δ*_*ij*_ as the Kronecker delta.

We are concerned with in-plane elastodynamic waves propagating along a linear microstructured grating such as that shown in figure 1. In particular, it is assumed that there are two natural length scales: (i) a short scale ℓ>0 characterizing the dimension of the microstructure and (ii) a long scale *L*>0 characterizing the overall size of the macrostructure (or macrocell). This being the case, it is natural to introduce a slow variable *X*=*x*_1_/*L* along the array and a fast variable ***ξ***=**x**/ℓ, where *ϵ*=ℓ/*L* and 0<*ϵ*≪1 such that *σ*_*ij*_(***x***)=*σ*_*ij*_(***ξ***,*X*) and *u*_*i*_(***x***)=*u*_*i*_(***ξ***,*X*). An important feature of the asymptotic approach is that we introduce these two scales in space and that they are now treated as independent and therefore this builds the mismatch of scales into the equations.

In these new variables, the elastodynamic wave equation is then expressed as

3.3 $$L^{-1}\sigma_{i1,X}+\ell^{-1}\sigma_{ij,j}+\rho \omega^{2}u_{i}=0,$$

where subscript commas followed by lower case letters indicate differentiation with respect to the fast variables *ξ*_*j*_ and subscript commas followed by *X* indicate differentiation with respect to the slow variable *X*. Subscript commas followed by multiple characters indicate successive differentiation. In the rescaled variables, the constitutive relation (3.2) is

3.4 $$\sigma_{ij}=\ell^{-1}\mu (u_{i,j}+u_{j,i})+\ell^{-1}\lambda \delta_{ij}u_{k,k}+L^{-1}\mu (u_{i,X}\delta_{1j}+u_{j,X}\delta_{1i})+L^{-1}\lambda \delta_{ij}u_{1,X}.$$

Combining the rescaled equations (3.3) and (3.4) yields the governing equation of time-harmonic deformation in fast and slow variables

3.5 $$\begin{array}{rl} & u_{i,jj}+u_{j,ij}+\Lambda \delta_{ij}u_{k,kj}+\Omega^{2}u_{i}+\epsilon [2u_{i,1X}+u_{1,iX}+u_{k,kX}\delta_{1i}\\ & \;+\Lambda (u_{k,kX}\delta_{1i}+u_{1,Xi})]+\epsilon^{2}[u_{i,XX}+u_{1,XX}\delta_{1i}+\Lambda u_{1,XX}\delta_{i1}]=0, \end{array}$$

where the non-dimensional frequency-squared *Ω*^2^=ℓ^2^*ρω*^2^/*μ* and the ratio *Λ*=*λ*/*μ* have been introduced.

### (a) General, high-frequency, homogenization

The form of (3.5) suggests the following anstatz for the displacement field and frequency:

3.6 $$u_{i}=\sum_{n=0}^{\infty }\epsilon^{n}u_{i}^{\left(n\right)},\;\Omega^{2}=\sum_{n=0}^{\infty }\epsilon^{n}\Omega_{n}^{2},$$

whence a hierarchy of equations in ascending orders of *ϵ* is obtained (there is no need to go beyond second order for the present theory):

3.7 $$\begin{array}{rl} O(1):\;\sigma_{ij,j}^{\left(0\right)}+\Omega_{0}^{2}u_{i}^{\left(0\right)} & =0, \end{array}$$

3.8 $$\begin{array}{rl} O(\epsilon ):\;\sigma_{ij,j}^{\left(1\right)}+\Omega_{0}^{2}u_{i}^{\left(1\right)} & =-\Omega_{1}^{2}u_{i}^{\left(0\right)}-2u_{i,1X}^{\left(0\right)}-u_{1,Xi}^{\left(0\right)}-u_{j,jX}^{\left(0\right)}\delta_{1i}-\Lambda (u_{k,kX}^{\left(0\right)}\delta_{i1}+u_{1,iX}^{\left(0\right)}) \end{array}$$

and

3.9 $$\begin{array}{rl} O(\epsilon^{2}):\;\sigma_{ij,j}^{\left(2\right)}+\Omega_{0}^{2}u_{i}^{\left(2\right)} & =-\Omega_{1}^{2}u_{i}^{\left(1\right)}-2u_{i,1X}^{\left(1\right)}-u_{1,Xi}^{\left(1\right)}-u_{j,jX}^{\left(1\right)}\delta_{1i}-\Lambda (u_{k,kX}^{\left(1\right)}\delta_{i1}+u_{1,iX}^{\left(1\right)})\\ & \;-\Omega_{2}^{2}u_{i}^{\left(0\right)}-u_{i,XX}^{\left(0\right)}-u_{1,XX}^{\left(0\right)}\delta_{1i}-\Lambda u_{i,XX}^{\left(0\right)}\delta_{1i}, \end{array}$$

where $\sigma_{ij}^{\left(n\right)}=u_{i,j}^{\left(n\right)}+u_{j,i}^{\left(n\right)}+\Lambda u_{k,k}^{\left(n\right)}\delta_{ij}$ and ***ξ***∈*S*, where *S* is the elementary cell which contains some microstructural element *S*_0_ (see figure 1, for example).

The RB waves travel along an infinite, periodic or quasi-periodic, array and decay exponentially in the directions perpendicular to the array. The approach here is to create a perturbation about standing wave solutions which are either in-phase or out-of-phase across the elementary cell (figure 1). In this case, the elementary cell is the infinite strip $S=\{\xi :|\xi_{1}|<1,|\xi_{2}|<\infty \}\setminus S_{0}$ which contains some microstructural element *S*_0_ with boundary ∂*S*_0_. The exterior boundaries of the cell are denoted by ∂*S*_±_={***ξ***:*ξ*_1_=±1} (see figure 1, for example). The in-phase and out-of-phase conditions on the edge of the cell are then

3.10 $$u_{i}{|}_{\partial S_{+}}=\pm u_{i}{|}_{\partial S_{-}}\;\mathrm{and}\;\sigma_{ij}n_{j}{|}_{\partial S_{+}}=\pm \sigma_{ij}n_{j}{|}_{\partial S_{-}},$$

with + (resp. −) for the in-phase (resp. out-of-phase) solutions. Here, *n*_*j*_ are the components of the outward pointing normal. As discussed in §2, we require that the microstructure is traction free for RB waves, and then

3.11 $$\sigma_{ij}n_{j}{|}_{\partial S_{0}}=0.$$

The hierarchy of equations (3.7)–(3.9) is then supplemented with the following boundary conditions on ∂*S*_0_:

3.12 $$\begin{array}{rl} O(1):\sigma_{ij}^{\left(0\right)}n_{j} & =0, \end{array}$$

3.13 $$\begin{array}{rl} O(\epsilon ):\;\sigma_{ij}^{\left(1\right)}n_{j} & =-u_{i,X}^{\left(0\right)}n_{1}-u_{j,X}^{\left(0\right)}n_{j}\delta_{i1}-\Lambda u_{1,X}^{\left(0\right)}n_{i} \end{array}$$

and

3.14 $$\begin{array}{rl} O(\epsilon^{2}):\;\sigma_{ij}^{\left(2\right)}n_{j} & =-u_{i,X}^{\left(1\right)}n_{1}-u_{j,X}^{\left(1\right)}n_{j}\delta_{i1}-\Lambda u_{1,X}^{\left(1\right)}n_{i}. \end{array}$$

Returning to the O(1) problem and assuming that $\Omega_{0}^{2}$ is a simple eigenvalue (eigenvalues of multiplicity greater than one will be discussed later in the context of low-frequency homogenization), the leading-order solution admits the decomposition

$${\text{u}}^{\left(0\right)}(\xi ,X)=f^{\left(0\right)}(X){\text{U}}^{\left(0\right)}(\xi ).$$

It initially appears counterintuitive that the long-scale behaviour reduces to a single scalar function *f*^(0)^(*X*): as we are working with the vector system of in-plane elasticity we might naturally expect two scalar functions analogous to the shear and compressional potentials. Nevertheless, the leading-order problem formally permits the aforementioned decomposition and the elastic high-frequency homogenization theory of [35] also generates a similar situation. As we shall see in §3*b*, two scalar functions emerge naturally from the asymptotic analysis near the repeated root at zero frequency, which yield the expected shear and compressional potentials and, hence, the two light-lines emerging from the origin in figure 2*b*.

In order to determine $\Omega_{1}^{2}$, the following equality, formed by multiplying the O(1) equation by $u_{i}^{\left(1\right)}$ and subtracting the O(ϵ) equation multiplied by $U_{i}^{\left(0\right)}$, is considered

3.15 $$\begin{array}{rl} & \int_{S}[\sigma_{ij,j}^{\left(0\right)}u_{i}^{\left(1\right)}-\sigma_{ij,j}^{\left(1\right)}U_{i}^{\left(0\right)}]\;d\xi =\Omega_{1}^{2}f^{\left(0\right)}(X)\int_{S}∥{\text{U}}^{\left(0\right)}{∥}^{2}\;d\xi \\ & \;+\frac{df^{\left(0\right)}}{dX}\int_{S}[2U_{i,1}^{\left(0\right)}+U_{1,i}^{\left(0\right)}+U_{j,j}^{\left(0\right)}\delta_{1i}+\Lambda (U_{k,k}^{\left(0\right)}\delta_{i1}+U_{1,i}^{\left(0\right)})]U_{i}^{\left(0\right)}\;d\xi . \end{array}$$

By means of integrating by parts, and use of the boundary conditions, all terms except those involving $\Omega_{1}^{2}$ vanish yielding *Ω*_1_=0. Hence, the O(ϵ) equation admits solutions of the form

$$u_{i}^{\left(1\right)}(\xi ,X)=U_{i}^{\left(1\right)}(\xi )\frac{df^{\left(0\right)}}{dX},$$

where **U**^(1)^ satisfies

3.16 $$\Sigma_{ij,j}^{\left(1\right)}+\Omega_{0}^{2}U_{i}^{\left(1\right)}=-2U_{i,1}^{\left(0\right)}-U_{1,i}^{\left(0\right)}-U_{j,j}^{\left(0\right)}\delta_{1i}-\Lambda \left(U_{k,k}^{\left(0\right)}\delta_{i1}+U_{1,i}^{\left(0\right)}\right),\;\mathrm{for}\;\xi \in S$$

subject to the boundary condition

3.17 $$\Sigma_{ij}^{\left(1\right)}n_{j}=-U_{i}^{\left(0\right)}n_{1}-U_{j}^{\left(0\right)}n_{j}\delta_{i1}-\Lambda U_{1}^{\left(0\right)}n_{i},\;\text{for}\;\xi \in \partial S_{0}$$

where we introduce the short-scale stress ***Σ***^(*n*)^(***ξ***) as $\Sigma_{ij}^{\left(n\right)}=U_{i,j}^{\left(n\right)}+U_{j,i}^{\left(n\right)}+\Lambda U_{k,k}^{\left(n\right)}\delta_{ij}$. The next-to-leading order vector **U**^(1)^ is not required for the determination of *f*^(0)^ and, hence, is irrelevant for our purposes here.

Moving to the second-order problem and forming a similar solvability condition as for the O(ϵ) case the following homogenized differential equation for the envelope function *f*^(0)^(*X*) is obtained

3.18 $$A\frac{d^{2}f^{\left(0\right)}}{dX^{2}}+B\Omega_{2}^{2}f^{\left(0\right)}(X)=0,$$

where

3.19 $$\begin{array}{rl} A & =\int_{S}\{2{\left(U_{1}^{\left(0\right)}\right)}^{2}+{\left(U_{2}^{\left(0\right)}\right)}^{2}+\Lambda {\left(U_{1}^{\left(0\right)}\right)}^{2}+U_{i,1}^{\left(1\right)}U_{i}^{\left(0\right)}+U_{1,i}^{\left(1\right)}U_{i}^{\left(0\right)}\\ & \;+\Lambda U_{i,i}^{\left(1\right)}U_{1}^{\left(0\right)}-U_{i}^{\left(1\right)}U_{i,1}^{\left(0\right)}-U_{i}^{\left(1\right)}U_{1,i}^{\left(0\right)}-\Lambda U_{1}^{\left(1\right)}U_{i,i}^{\left(0\right)}\}\;d\xi \end{array}$$

and

3.20 $$B=\int_{S}∥{\text{U}}^{\left(0\right)}{∥}^{2}\;d\xi .$$

On the long-scale, the effective medium is then entirely characterized by (3.18) which is equivalent to that of a one-dimensional string of stiffness *T*=*A*/*B*. It is emphasized that the problem for the scalar envelope function (3.18) is cast entirely on the long-scale, with the short-scale properties of the microstructure encoded in the *T*-values. The overall leading-order behaviour is the rapid short-scale oscillations described by the leading-order eigenvector **U**^(0)^, modulated by the long-scale oscillations of *f*^(0)^. Indeed, for waves of frequency *Ω* propagating along the diffraction grating, the amplitude of the short-scale oscillations are modulated by a long-scale oscillation of wavelength $\lambda =2\pi \sqrt{T/(\Omega^{2}-\Omega_{0}^{2})}$.

The homogenized differential equation (3.18) can be used to obtain the asymptotic dispersion relation in the vicinity of the standing wave frequencies at *k*=*π*/2. The Bloch–Floquet quasi-periodicity condition $\text{u}{|}_{\partial S_{-\ell }}=\text{u}{|}_{\partial S_{+\ell }}\;e^{i2k\ell }$ requires that the solution be anti-periodic with respect to *ξ*_1_ at *k*=*π*/2. As **U**^(0)^(***ξ***) is anti-periodic by construction, we choose $f^{\left(0\right)}(X)=\exp(i[\pi /2-k]X/\epsilon )$ which, after substitution into (3.18), yields

3.21 $$\Omega_{2}^{2}=\epsilon^{-2}T{\left(\frac{\pi }{2}-k\right)}^{2},$$

whence the asymptotic dispersion equation is

3.22 $$\Omega ∼\Omega_{0}+\frac{T}{2\Omega_{0}}{\left(\frac{\pi }{2}-k\right)}^{2}\;\text{as}\;|k|\to \frac{\pi }{2}.$$

### (b) Classical, low-frequency, homogenization

In the low-frequency regime, that is when $\Omega^{2}=O(\epsilon^{2})$, the above procedure is still valid but there are some important technical differences. The boundary value problem for the leading order is

3.23 $$\sigma_{ij,j}^{\left(0\right)}=0,\;\mathrm{for}\;\xi \in S\;\mathrm{and}\;\sigma_{ij}^{\left(0\right)}n_{j}=0,\;\mathrm{for}\;\xi \in \partial S_{0},$$

and periodic conditions are imposed on the lateral boundaries of the elementary strip. The leading-order boundary value problem has an eigenvalue *Ω*_0_=0 of multiplicity two and, hence, admits a solution of the form

3.24 $$u_{i}^{\left(0\right)}=f^{\left(0,\ell \right)}(X)U_{i}^{\left(0,\ell \right)},$$

where repeated indices are summed over and $U_{i}^{\left(0,\ell \right)}$ are constant. For simplicity and without loss of generality, we take $U_{i}^{\left(0,\ell \right)}=\delta_{i\ell }$. The next-to-leading order problem is

3.25 $$\begin{array}{rl} \sigma_{ij,j}^{\left(1\right)} & =0,\;\mathrm{for}\;\xi \in S \end{array}$$

and

3.26 $$\begin{array}{rl} \sigma_{ij}^{\left(1\right)}n_{j} & =-\frac{df^{\left(0,\ell \right)}}{dX}(\delta_{i\ell }\delta_{1j}+\delta_{j\ell }\delta_{1i}+\Lambda \delta_{ij}\delta_{1\ell })n_{j},\;\text{for}\;\xi \in \partial S_{0}, \end{array}$$

which admits solutions of the form

3.27 $$u_{i}^{\left(1\right)}(X)=U_{i}^{\left(1,\ell \right)}\frac{df^{\left(0,\ell \right)}}{dX}.$$

Finally, the spectral parameter appears in the second-order problem

3.28 $$\begin{array}{rl} \sigma_{ij,j}^{\left(2\right)} & =-\Omega_{2}^{2}f^{\left(0,\ell \right)}U_{i}^{\left(0,\ell \right)}\delta_{i\ell }-\frac{d^{2}f^{\left(0,\ell \right)}}{dX^{2}}[2U_{i,1}^{\left(1,\ell \right)}+U_{1,i}^{\left(1,\ell \right)}+U_{k,k}^{\left(1,\ell \right)}\delta_{1i}\\ & \;+\Lambda (U_{k,k}^{\left(1,\ell \right)}\delta_{1i}+U_{1,i}^{\left(1,\ell \right)})+\delta_{\ell i}+\delta_{\ell 1}\delta_{i1}(1+\Lambda )],\;\text{for}\;\xi \in S \end{array}$$

and

3.29 $$\begin{array}{rl} \sigma_{ij}^{\left(2\right)}n_{j} & =-\frac{d^{2}f^{\left(0,\ell \right)}}{dX^{2}}(U_{i}^{\left(1,\ell \right)}\delta_{1j}+U_{j}^{\left(1,\ell \right)}\delta_{1i}+\Lambda \delta_{ij}U_{1}^{\left(1,\ell \right)})n_{j},\;\text{for}\;\xi \in \partial S_{0}. \end{array}$$

The solvability condition yields the following homogenized differential equation for *f*^(0,ℓ)^(*X*):

3.30 $$T_{\ell m}\frac{d^{2}f^{\left(0,\ell \right)}}{dX^{2}}+\Omega_{2}^{2}f^{\left(0,\ell \right)}=0,$$

where

3.31 $$T_{\ell m}=\delta_{\ell m}+\delta_{1\ell }\delta_{1m}+\Lambda \delta_{1\ell }\delta_{1m}+∥S{∥}^{-1}\int_{S}[U_{m,1}^{\left(1,\ell \right)}+U_{1,m}^{\left(1,\ell \right)}+\Lambda \delta_{1m}U_{k,k}^{\left(1,\ell \right)}]\;d\xi ,$$

and $∥S∥=\int_{S}d\xi$. For localized modes, that is for $U_{j,k}^{\left(i,\ell \right)}\to 0$ as $\xi_{2}\to \infty$, the final term vanishes yielding

3.32 $$T_{\ell m}=\delta_{\ell m}+\delta_{1\ell }\delta_{1m}+\Lambda \delta_{1\ell }\delta_{1m}.$$

Hence, returning to dimensional form, the homogenized continuum has two characteristic wave speeds ($c_{ij}=\sqrt{\mu T_{ij}/\rho }$)

3.33 $$c_{11}=\sqrt{\frac{\lambda +2\mu }{\rho }}\;\mathrm{and}\;c_{22}=\sqrt{\frac{\mu }{\rho }},$$

corresponding to compressional and shear waves, which yields two light-lines (e.g. figure 2*b*). Moreover, the diagonal entries of the tensor **T** can be identified with the P-wave modulus (*T*_11_) and the shear modulus (*T*_22_); the off-diagonal terms of **T** vanish. Thus, the homogenized behaviour of the elastodynamic system is governed by two scalar functions (alternatively a single vector function with two components), as expected [27].

## 4. An infinite array of cylindrical voids

As an example, we apply the high-frequency asymptotic procedure to an infinite line of traction-free cylindrical voids, as shown in figure 1. The material and geometrical parameters used are *μ*=1, *λ*=1.5 (hence $\nu =\frac{1}{3}$), *ρ*=1, ℓ=1, and the radius of the voids *S*_0_ is 0.8. This choice of radius is made because it is very close to the radius at which the fourth mode appears (cf. figure 3*b*). There exist five RB-like localized modes below the compressional light-line; the dispersion curves for each mode are shown in figure 3*a*. Figure 3*b* shows the variation in standing wave frequencies with respect to increasing radius of the voids. The dashed line indicates the intersection of the shear light-line with the line *k*=*π*/2 where we expect to see standing waves. For the radius chosen (*r*=0.8), figure 3*b* indicates that there are four RB-type modes underneath the shear light-line. For smaller radii, the number of modes decreases. It is interesting to note that the fourth RB mode emerges as we reach the chosen radius of *r*=0.8; that is, *r*≈0.8 is the critical radius at which a fourth RB mode emerges and descends from the shear light-line (cf. figure 3*b*). The dispersion curve is constrained to remain below the shear light-line and hence appears to be very short in figure 3*a*. Moreover, as the fourth mode lies very close to the light-line, it is relatively weakly localized. Indeed, figure 10 illustrates that the RB wave is shear dominated and the leading order fields resemble a bulk elastic wave. Although it may appear from figure 10*b*,*c* that the field does not decay in the *x*_2_ direction it, in fact, does but very slowly. A similar effect is observed in the scalar case [20].

The two rays emanating from the origin have slopes as defined in (3.33) and represent the shear and compressional light-lines, which are obtained from the low frequency asymptotics as described in §3*b*. Moving to the end of the Brillouin zone (*k*=*π*/2), where the displacement fields in neighbouring cells are out of phase with each other, we find five localized modes at frequencies away from *Ω*=0. As can be seen from figure 3*a*, the asymptotic procedure captures the behaviour of the dispersion curves very well in the vicinity of the standing wave frequencies.

The standing wave frequencies, together with the associated *T*-values, computed using the high-frequency asymptotic procedure as described in §3 are detailed in table 1. The *T*-value for the fourth mode is omitted as it emerges very close to the shear light-line. The *T*-values represent the stiffness of the homogenized material, which replaces the array of voids on the long-scale.

**Table 1.** The standing wave frequencies, homogenized *T*-values and PS-ratios, cf. equation (4.1), for the five localized modes shown in figure 3*a*. The *T*-value for the fourth mode is omitted as the curve emerges very close to the shear light-line.

frequency (*Ω*_0_)	homogenized *T*-value	PS-ratio *χ*
1.220	−2.474	0.267
1.375	−16.27	0.333
1.486	14.27	0.192
1.569	—	0.0611
2.365	−5.868	1.33

The implementation uses a bespoke algorithm developed for the commercial finite-element package Comsol Multiphysics in order to solve the required eigenvalue problems for **U**^(0)^(***ξ***) and **U**^(1)^(***ξ***) and, hence, obtain the *T*-values shown in table 1. It is worth mentioning that other appropriate methods do exist, which could be used to investigate the problem at hand. In particular, an alternative, semi-analytical method, would be to use multipole expansions about the centre of the circular voids in a similar approach to that used in scattering problems for elasticity (e.g. [25,26]). The use of multipole expansions may also lead to further simplification of the expressions for the *T*-values.

**Figure 5.**
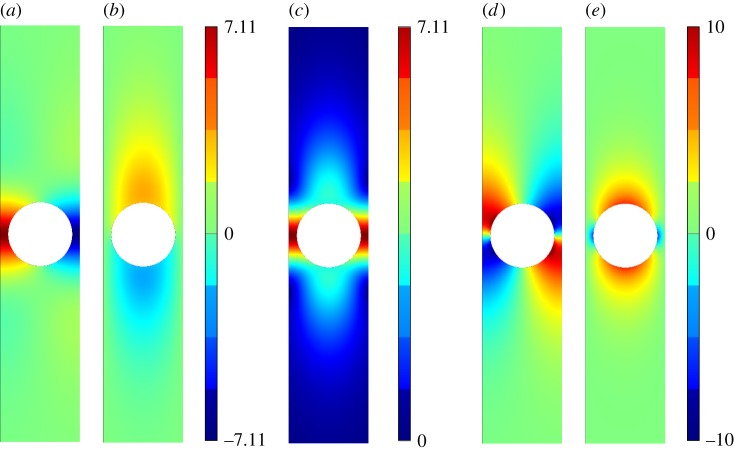
The displacement field (*a*–*c*) together with the shear (*d*) and pressure potentials (*e*) for the first mode. (*a*) $U_{1}^{\left(0\right)}$, (*b*) $U_{2}^{\left(0\right)}$, (*c*) ∥**U**^(0)^∥, (*d*) *ψ*^(0)^ and (*e*) *φ*^(0)^. (Online version in colour.)

**Figure 6.**
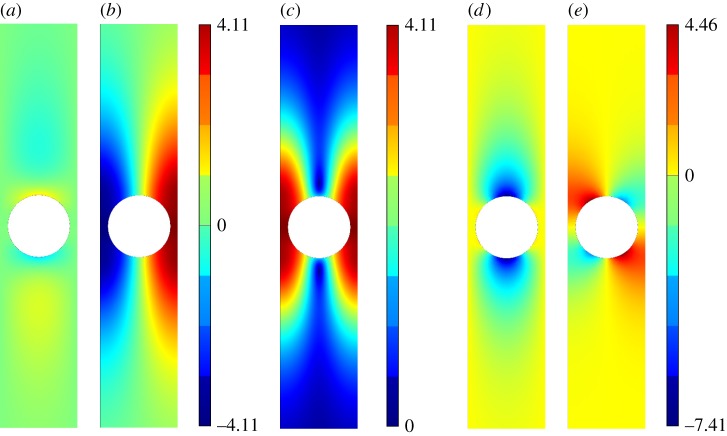
The displacement field (*a*–*c*) together with the shear (*d*) and pressure potentials (*e*) for the second mode. We draw the reader’s attention to the asymmetry of the colour scale in (*d*),(*e*). (*a*) $U_{1}^{\left(0\right)}$, (*b*) $U_{2}^{\left(0\right)}$, (*c*) ∥**U**^(0)^∥, (*d*) *ψ*^(0)^ and (*e*) *φ*^(0)^. (Online version in colour.)

**Figure 7.**
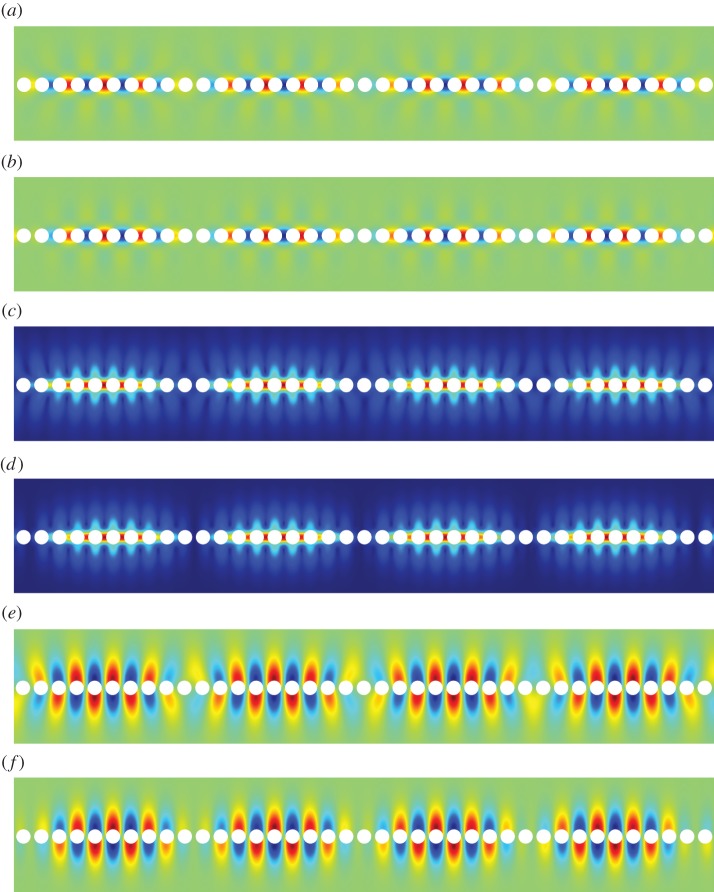
The first RB wave propagating along the diffraction grating. Mode one: (*a*) numerics: $U_{1}^{\left(0\right)}$, (*b*) asymptotics: $U_{1}^{\left(0\right)}$, (*c*) numerics: ∥**U**^(0)^∥, (*d*) asymptotics: ∥**U**^(0)^∥, (*e*) numerics: $U_{2}^{\left(0\right)}$ and (*f*) asymptotics: $U_{2}^{\left(0\right)}$. (Online version in colour.)

**Figure 8.**
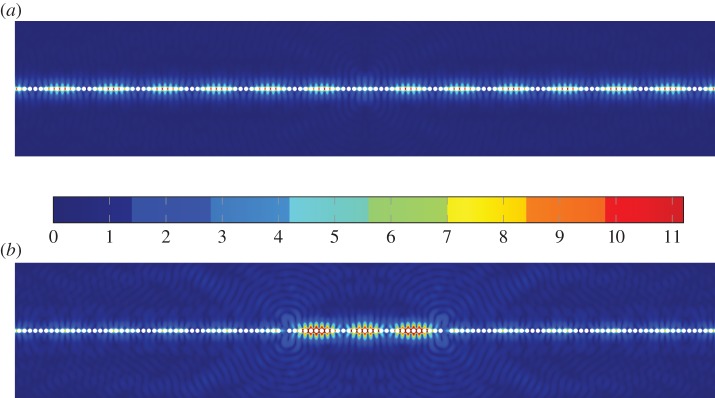
Plots of ∥**u**(**x**)∥ illustrating the confinement of a RB mode by a system of two defects. In this case, the defects are created by removing two voids from the array. It is remarked that the field amplitude is (*a*) perfect diffraction grating and (*b*) diffraction grating with defects in this case the field amplitude inside the confinement region is approximately an order of magnitude greater than that outside the confinement region. (Online version in colour.)

**Figure 9.**
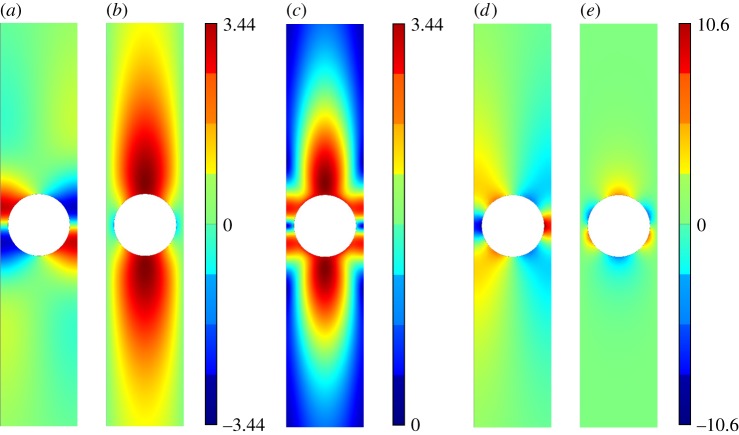
The displacement field (*a*–*c*) together with the shear (*d*) and pressure potentials (*e*) for the third mode. (*a*) $U_{1}^{\left(0\right)}$, (*b*) $U_{2}^{\left(0\right)}$, (*c*) ∥**U**^(0)^∥, (*d*) *ψ*^(0)^ and (*e*) *φ*^(0)^. (Online version in colour.)

**Figure 10.**
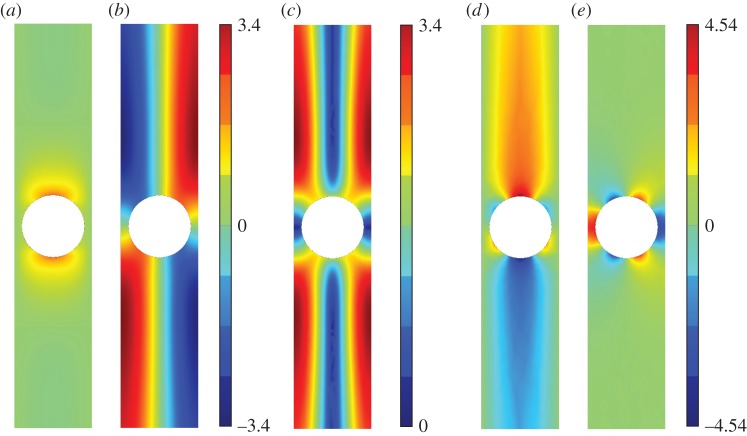
The displacement field (*a*–*c*) together with the shear (*d*) and pressure potentials (*e*) for the fourth mode. (*a*) $U_{1}^{\left(0\right)}$, (*b*) $U_{2}^{\left(0\right)}$, (*c*) ∥**U**^(0)^∥, (*d*) *ψ*^(0)^ and (*e*) *φ*^(0)^.(Online version in colour.)

**Figure 11.**
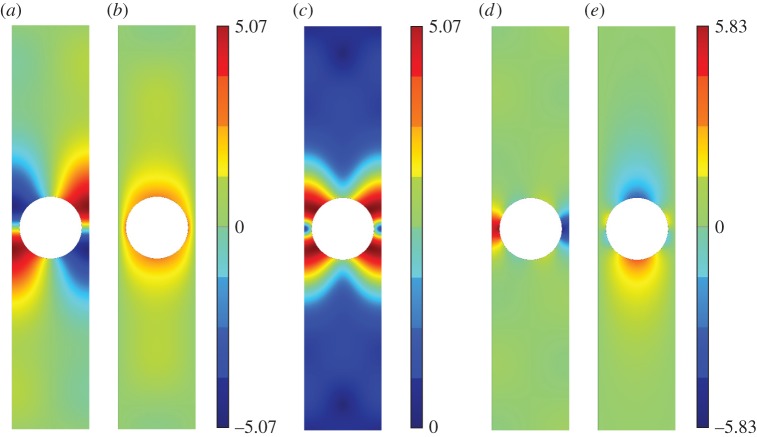
The displacement field (*a*–*c*) together with the shear (*d*) and pressure potentials (*e*) for the fifth mode. (*a*) $U_{1}^{\left(0\right)}$, (*b*) $U_{2}^{\left(0\right)}$, (*c*) ∥**U**^(0)^∥, (*d*) *ψ*^(0)^, (*e*) *φ*^(0)^. (Online version in colour.)

Figures 5–11 show the leading-order displacement fields **U**^(0)^(***ξ***), together, with the leading-order shear *ψ*^(0)^(***ξ***) and compressional *φ*^(0)^(***ξ***) potentials. It is clear from figures 5–11 that, although the problem is fully coupled, in some cases the shear potential is larger than the compressional potential and vice versa. Also shown in table 1 is the compressional to shear ratio *χ*, which we introduce as

4.1 $$\chi =\left[\int_{S}|\varphi^{\left(0\right)}{|}^{2}\;d\xi \right]\;{\left[\int_{S}|\psi^{\left(0\right)}{|}^{2}\;d\xi \right]}^{-1}.$$

The *χ*-values in table 1 further emphasize that the problem is a fully coupled problem of vector elasticity with none of the modes being entirely shear or compressional in nature. Nevertheless, we observe that modes close to the shear light-line (i.e. the first four modes) are dominated by the shear potential. However, it should be noted that even in cases where *χ* differs significantly from unity, the problem remains fully coupled through the boundary conditions on the inclusion ∂*S*_0_ and the edges of the elementary cell ∂*S*_±_ (via conversion from pressure to shear and vice versa). Moreover, the reader should bear in mind that the displacement field is obtained from linear combinations of the partial derivatives of the potentials, rather than the potentials themselves.

### (a) The macroscopic behaviour

Figures 5–11 illustrate the short-scale behaviour of the localized RB modes at the level of the microstructure. The long-scale macroscopic behaviour of the RB modes is governed by the homogenized equation (3.30), which admits solutions of the form

4.2 $$f^{\left(0\right)}(X)\propto \exp\;\left(-P.V.\sqrt{\frac{\Omega_{0}^{2}-\Omega^{2}}{T}}|X|\right),$$

where *Ω*_0_ is the standing wave frequency, P.V. denotes the principal value, and the negative sign is chosen to prevent growth of the macroscopic field at infinity. Clearly, the long-scale behaviour depends on the sign of the argument of the square root: if $(\Omega_{0}^{2}-\Omega^{2})/T$ is negative, then the macroscale envelope function oscillates with a wavelength of $2\pi \sqrt{T/(\Omega_{0}^{2}-\Omega^{2})}$. The overall response is thus, rapid oscillations of frequency *Ω*_0_ on the short-scale, modulated by a wave with beat frequency $\sqrt{\Omega_{0}^{2}-\Omega^{2}}$. On the other hand, if $(\Omega_{0}^{2}-\Omega^{2})/T$ is positive then the macroscale envelope function exponentially decays with increasing *X*. So while the short-scale oscillations, as defined by **U**^(0)^(***ξ***) persist, their amplitude decays exponentially.

Physically, the two cases can be thought of as a perturbation away from the standing wave frequency *Ω*_0_ into the pass (resp. stop) band for the particular mode when $(\Omega_{0}^{2}-\Omega^{2})/T$ is negative (resp. positive). It is emphasized that the beat frequency of the macroscale envelope function depends both, on the perturbation away from the standing wave frequency, and the microscale properties encoded on the dynamically homogenized stiffness *T*.

**Figure 12.**
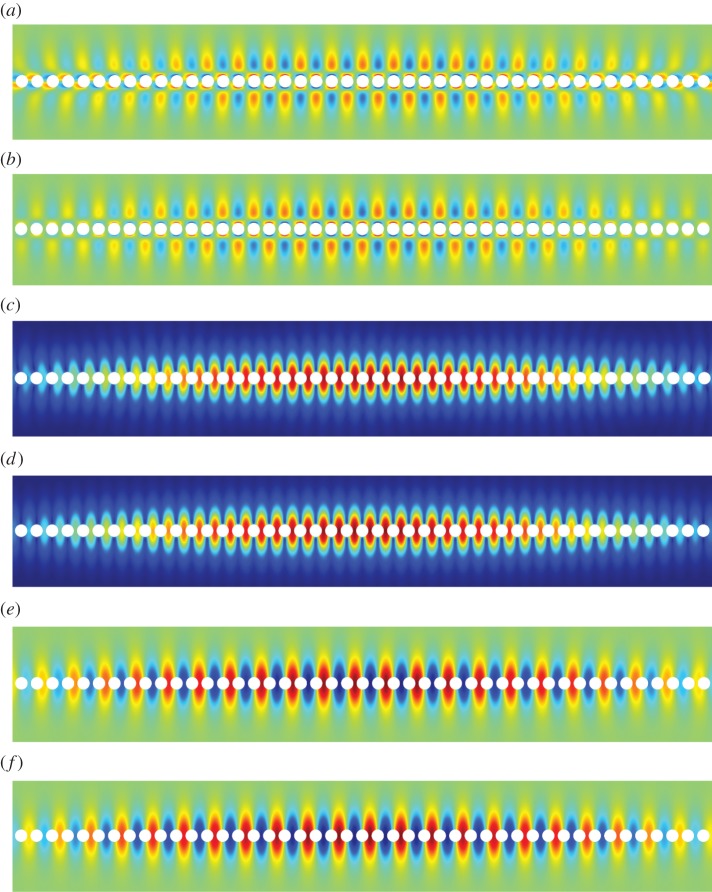
The second RB wave propagating along the diffraction grating. Mode two: (*a*) numerics: $U_{1}^{\left(0\right)}$, (*b*) asymptotics: $U_{1}^{\left(0\right)}$, (*c*) numerics: ∥**U**^(0)^∥, (*d*) asymptotics: ∥**U**^(0)^∥, (*e*) numerics: $U_{2}^{\left(0\right)}$ and (*f*) asymptotics: $U_{2}^{\left(0\right)}$. (Online version in colour.)

**Figure 13.**
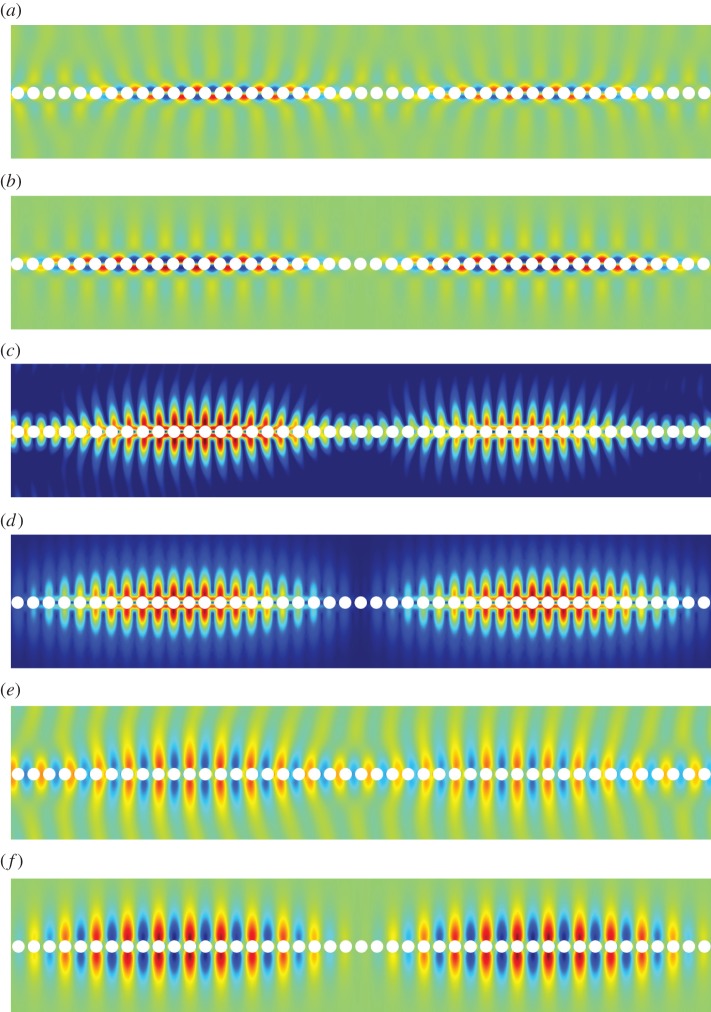
The third RB wave propagating along the diffraction grating. Mode three: (*a*) numerics: $U_{1}^{\left(0\right)}$, (*b*) asymptotics: $U_{1}^{\left(0\right)}$, (*c*) numerics: ∥**U**^(0)^∥, (*d*) asymptotics: ∥**U**^(0)^∥, (*e*) numerics: $U_{2}^{\left(0\right)}$ and (*f*) asymptotics: $U_{2}^{\left(0\right)}$. (Online version in colour.)

Figures 7–13 illustrate the overall behaviour of the RB modes. The illustrative computations were produced using the commercial finite-element software Comsol Multiphysics and simulated RB waves, excited by a remote source, and propagating along an infinite array of traction-free cylindrical voids. Figures 7, 12 and 13 correspond to modes one, two and three, respectively; parts (*a*), (*c*) and (*e*) are the results of the numerical simulations, while parts (*b*), (*c*) and (*f*) are obtained from the high-frequency homogenization scheme. It is clear from these figures, firstly that in-plane elastic RB modes exist, and secondly that the high-frequency homogenization scheme captures their behaviour very well. Both, the rapid short-scale oscillations, and the macroscale envelope functions are accurately reproduced by the homogenization scheme.

In figure 7, the excitation frequency is *Ω*=1.193, which corresponds to a long-scale wavelength of 39.15, or approximately 19.58 elementary cells. The wavelength of the envelope function as observed in figure 7 is actually 19.57; the factor of one half arises as both *f*^(0)^(*X*) and −*f*^(0)^(*X*) are excited by the remote source. In figure 12, the excitation frequency is *Ω*=1.370, resulting in a smaller perturbation away from the standing wave frequency *Ω*_0_=1.374 and, hence, a longer macroscopic wavelength. Plots of the long-scale behaviour for the higher frequency modes are omitted for brevity. Illustrative examples of perturbations into local stop bands (i.e. for $(\Omega_{0}^{2}-\Omega^{2})/T>0$) are also omitted as propagating RB waves are of primary interest.

## 5. Concluding remarks

The recent proposal of geometrically induced (or spoof) SPP with structured metal interfaces [7] has renewed the interest of physicists and mathematicians alike in RB waves. The spoof SPP concept was first realized experimentally on planar geometries [38,39], but it has been also extended to wedge diffraction gratings [40], where strong confinement of electromagnetic waves has been demonstrated in the terahertz regime. Potential applications of RB waves in plasmonics lie in photovoltaics via photo-thermal effects, but recent experiments on light and sound interplay through Brillouin scattering in arrays of fibres with defects [41,42] suggest that the existence of RB waves in elastodynamics might open new vistas in fast opto-elastic switches.

The most significant outcome of this paper is the demonstration that RB waves do exist for diffraction gratings in the setting of fully coupled (in-plane) vector elasticity. Despite significant interest for scalar problems (acoustics, flexural waves, polarized electromagnetic waves, etc.) and their numerous applications, this paper is the first study of RB waves for elastic diffraction gratings in the scientific literature. By couching this study in the language of homogenization, we were also able to develop an efficient asymptotic homogenization theory for RB waves. Indeed, owing to the large number of spurious modes generated by the numerical implementation of perfectly matched layers and infinite-element domains, there are significant computational challenges in extracting RB waves numerically. However, the behaviour of the RB waves can be conveniently obtained using the asymptotic procedure developed herein. Moreover, using the homogenization theory, long diffraction gratings which are computationally expensive to simulate (such as those illustrated in figures 7–13) can be replaced by an effective continuum as detailed in §3, whence the two-dimensional diffraction grating problem reduces to a one-dimensional problem on a line-segment. Figures 7–13 are a testament to the efficacy of this approach.

Beyond efficient numerical modelling, one can envisage many practical applications of elastic RB waves. For example, the long-scale wavelength can be altered by the introduction of a perturbation or defect in the diffraction grating and this effect could be used to filter or guide waves of specific frequency: see figure 8, wherein the removal of two voids within the grating leads to a confinement of the RB wave in the defect so created. It should be noted that the length of the defect can be adjusted to support a larger number *N* of beats of the RB wave, i.e. one needs to remove one inclusion every 8*N* to 9*N* inclusions. Such a localization result, in conjunction with recent advances in light and heat confinement in phononic crystals [42], suggests a whole new range of applications with elastic gratings. From the point of view of non-destructive evaluation, one could infer properties of a diffraction grating (e.g. weldment or riveted joint) from the scattered waves; waves could even be guided along structured joints during ultrasonic testing.

Similar problems have been considered, by Thompson and co-workers [43,44], for acoustical diffraction gratings. In [43], Linton *et al.* studied the excitation of RB waves along semi-infinite diffraction gratings consisting of sound-hard scatterers. In the later paper, Thompson & Linton [44] developed the earlier analysis to study scattering by clusters of defects in infinite arrays of Neumann scatterers; in this case, the defects were formed by removing a finite number of scatters from the array. There the authors found that the defects excited RB waves and, moreover, that the scattered RB waves could be trapped between two widely spaced scatterers.

More fundamentally, the existence of RB waves for elastic diffraction gratings raises interesting questions with regard to other wave phenomena, which exist in scalar problems but have yet to be studied in elasticity. Given its wide applicability (see [20,31,32,34,35,45] among others), the high-frequency homogenization procedure would seem to be an excellent tool with which to further examine possible correspondences between phenomena across a wide range of physical systems.

## Footnotes

1 It is remarked that RB waves exist on infinite or semi-infinite gratings and, as such, L→∞. Nevertheless, in a practical setting, the experimental diffraction grating would necessarily be large but finite leading to a large but finite *L*.
